# Nano-scale characterization of iron-carbohydrate complexes by cryogenic scanning transmission electron microscopy: Building the bridge to biorelevant characterization

**DOI:** 10.1016/j.heliyon.2024.e36749

**Published:** 2024-08-24

**Authors:** Reinaldo Digigow, Michael Burgert, Marco Luechinger, Alla Sologubenko, Andrzej J. Rzepiela, Stephan Handschin, Amy E. Barton Alston, Beat Flühmann, Erik Philipp

**Affiliations:** a CSL Vifor, Flughofstrasse 61, CH-8152, Glattbrugg, Switzerland; b Scientific Center for Optical and Electron Microscopy, ScopeM, ETH Zürich, 8093, Zürich, Switzerland

**Keywords:** Iron deficiency anemia, Nanomedicines, Nanoparticles (NPs), Non-biological complex drugs (NBCDs), Iron-carbohydrate complexes, Physicochemical characterization of nanomedicines, Cryo-scanning transmission electron microscopy (cryo-STEM), Cryo-transmission electron microscopy (cryo-TEM)

## Abstract

Iron deficiency and iron deficiency anemia pose significant health challenges worldwide. Iron carbohydrate nanoparticles administered intravenously are a mainstay of treatment to deliver elemental iron safely and effectively. However, despite decades of clinical use, a complete understanding of their physical structure and the significance for their behavior, particularly at the nano-bio interface, is still lacking, underscoring the need to employ more sophisticated characterization methods. Our study used cryogenic Scanning Transmission Electron Microscopy (cryo-STEM) to examine iron carbohydrate nanoparticle morphology. This method builds upon previous research, where direct visualization of the iron cores in these complexes was achieved using cryogenic Transmission Electron Microscopy (cryo-TEM). Our study confirms that the average size of the iron cores within these nanoparticles is approximately 2 nm across all iron-based products studied. Furthermore, our investigation revealed the existence of discernible cluster-like morphologies, not only for ferumoxytol, as previously reported, but also within all the examined iron-carbohydrate products. The application of cryo-STEM for the analyses of product morphologies provides high-contrast and high-resolution images of the nanoparticles, and facilitates the characterization at liquid nitrogen temperature, thereby preserving the structural integrity of these complex samples. The findings from this study offer valuable insights into the physical structure of iron-carbohydrate nanoparticles, a crucial step towards unraveling the intricate relationship between the structure and function of this widely used drug class in treating iron deficiency. Additionally, we developed and utilized the self-supervised machine learning workflow for the image analysis of iron-carbohydrate complexes, which might be further expanded into a useful characterization tool for comparability studies.

## Graphical abstract

**Figure:**
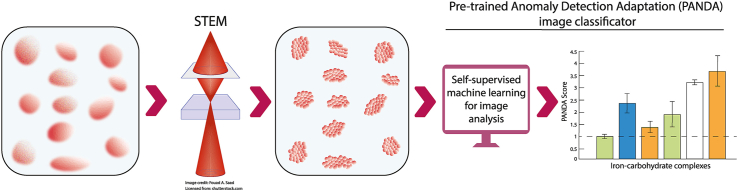

## 1 Introduction

Iron is a crucial element for erythropoiesis and formation of red blood cells. It is also essential for many cellular functions, including DNA synthesis, glycolysis, fatty acid oxidation, and mitochondrial energy metabolism [1,2]. Iron deficiency is a global health problem that affects more than one billion people worldwide [3]. Many diseases are associated with iron deficiency and iron deficiency anemia, including cancer, menorrhagia, postpartum hemorrhage, chronic kidney disease, heart failure, and inflammatory bowel disease [4,5]. Iron supplementation remains a mainstay for the treatment of iron deficiency with or without anemia. However, oral iron salts generally have limited efficacy due to poor absorption and adverse gastrointestinal effects leading to poor adherence for certain patient groups [6]. Thus, administration of iron intravenously was introduced as an alternative to oral treatment. However, early studies evaluating the intravenous (IV) administration of iron salts or “naked” polynuclear ferric oxyhydroxides demonstrated significant and unacceptable toxicity risks [7]. The development of iron-carbohydrate complexes allowed for safer and more effective administration of intravenous iron. The clinically available IV iron-carbohydrate complexes are nanoparticles composed of polynuclear iron cores with a carbohydrate ligand protecting the polynuclear core from immediate dissolution in plasma after drug administration and allowing the iron to be incorporated into the mononuclear phagocytic system (MPS) [8,9]. The design of such iron-carbohydrate nanoparticles was inspired by the molecular conformation of endogenous serum ferritin, which stores ferric iron by forming iron-protein complexes and homeostatically releases iron. The carbohydrate ligands dictate their pharmacokinetic profile and tolerability of the product [8]. Several iron-carbohydrate complexes are available on the market. Clinical trials have demonstrated that these products effectively furnish their pharmacologic effect of iron delivery evidenced by the increase in hemoglobin and serum ferritin concentrations [6].

The current understanding of the mechanism of action of the iron-carbohydrate complexes relies on the hypothesis that after the intravenous administration, the nanoparticles are taken up by circulating macrophages and then delivered to the MPS, where they are biodegraded by a yet to be elucidated process. In animal models, each iron-carbohydrate product demonstrates its own unique iron biodistribution profile [9]. The pharmacokinetic profile of total serum iron in humans also varies for different IV iron-carbohydrate products with different carbohydrate ligands [10]. Moreover, they differ in terms of the content and release of labile iron as well as the generation of low molecular weight iron species [[11], [12], [13], [14], [15]]. These nanoparticles have complex physicochemical properties, such as their morphologies and internal structures which are believed to be relevant contributors to the pharmaceutical quality and the pharmacological properties. However, their critical quality attributes (CQAs) are still not fully established [16,17]. Due to the high complexity of intravenous iron carbohydrate products, they cannot be yet fully characterized by physicochemical methods. Therefore, they are considered non-biological complex drugs (NBCD) [18,19], and FDA has classified them as complex drugs [20]. To facilitate a deeper understanding of the structure-property relationships of iron-carbohydrate complexes, scientists [21] and the regulatory agencies [[22], [23], [24]] have suggested the use of orthogonal characterization techniques, which can facilitate a better understanding of the *in vivo* behavior of these nanomedicines.

Transmission electron microscopy (TEM) is a powerful technique for complex material characterization on the nano- and micro-scales. TEM is routinely employed to provide morphological, structural, and chemical information from the same volume of a specimen. Since it uses the high-energy electron beam strongly interacting with atoms, electron radiation is prone to induce changes in the material state, such as phase transformation, phase decomposition and specimen contamination [[25], [26], [27]]. Thus, care must be taken to maintain the material in its most native state during the TEM specimen preparation and studies. The discrepancies in the publications reporting the iron-core size and the overall morphology of the iron-carbohydrate complexes from TEM studies have been the subject of extensive debate and can likely be attributed to the artifacts due to the electron beam induced material change [11,13,[28], [29], [30]]. Moreover, the room temperature TEM specimen preparation protocols can also promote agglomeration or coalescence of the nanoparticles. Thus, the ambient conditions during TEM specimen preparation and studies are the main source of the artifacts [31,32].

Y. Wu et al. [32] reported a direct visualization of the iron cores of iron-carbohydrate complexes in their most native state by employing cryogenic transmission electron microscopy (cryo-TEM) involving the vitrification of the studied material and performing the TEM analyses at liquid nitrogen temperature using a dedicated TEM specimen holder. The work clearly demonstrated that the commonly employed room temperature TEM specimen preparation techniques led to nanoparticle agglomeration biasing the morphological analyses of the materials. The cryo-TEM study of the same products prepared by preserving the iron-carbohydrate nanoparticles in their native, frozen-hydrated, and undiluted state revealed a spherical morphology of the iron cores with a narrow size distribution and an average size of 2 nm. Interestingly, ferumoxytol (FMX) was found to possess a cluster-like morphology composed of multiple iron cores combined in one cluster, which is consistent with its larger hydrodynamic size (approximately 25 nm) as determined by dynamic light scattering (DLS) analysis. Conversely, the cluster-like morphology was not observed for the rest of the products (*e.g.* iron sucrose (IS), sodium ferric gluconate (SFG), and low molecular weight iron dextran (LMWID)) studied. Previous cryo-TEM images showed the morphology of individual NPs (composed of only one iron core), with an average size of 2 nm [32]. This contradicts the reported DLS values, which indicated hydrodynamic sizes ranging from 8 to 12 nm. These hydrodynamic sizes likely represent the size of a cluster (i.e. composed of multiple iron cores arranged within the cluster) rather than the iron core alone [11,13,33]. Consequently, more advanced techniques such as STEM (employed using a cryo-setup), Small-angle X-ray scattering (SAXS) and Small-angle neutron scattering (SANS) (both used on specimens at ambient conditions), etc., are necessary for the characterization of these materials in their pristine state. Advancing these techniques with pristine materials will be key to enhance our understanding of iron-carbohydrate complexes at the nano-bio interface when evaluated in biological matrices [21,34].

In the present study, we employed cryogenic transmission electron microscopy (cryo-TEM), which operates using a parallel electron beam illumination, and scanning transmission electron microscopy (cryo-STEM), employing a conical, finely focused electron probe moving over the specimen region and illuminating a very small volume of it at a time, to study six commercially available iron-carbohydrate complexes (see details of the products in Table S1). The main advantage of cryo-STEM imaging is a considerably higher image contrast when compared to the cryo-TEM data [35], which in turn enables the higher resolution of the image features, clusters, and Fe-containing cores.

The goal of these studies, both cryo-TEM and cryo-STEM, was to resolve the discrepancy of previously reported size differences between DLS and cryo-TEM data by identifying the true morphology of the nanoparticulate iron carbohydrate complexes and their individual building blocks. Further, we aimed to develop the appropriate analytical criteria to facilitate the characterization of iron-carbohydrate complexes using machine learning. This study intents to provide fundamental insights into the structure of nanoparticulate iron carbohydrate complexes but it is not meant or does produce any direct conclusions about the *in-vivo* or clinical aspects of these products in relationship to the cryo-STEM findings.

## 2 Material and methods

### 2.1 Materials

Iron sucrose (IS) (20 mg Fe/mL; CSL Vifor, St. Gallen, Switzerland), ferric carboxymaltose (FCM) (50 mg Fe/mL; CSL Vifor, St. Gallen, Switzerland), low molecular weight iron dextran (LMWID) (50 mg Fe/mL; Teva, Mörfelden-Walldorf, Germany), Iron isomaltoside 1000 (IIM) (100 mg Fe/mL; Pharmacosmos, Holbaek, Denmark), Sodium ferric gluconate (SFG) (12.5 mg Fe/mL; Sanofi-Aventis, Frankfurt, Germany), and Ferumoxytol (FMX) (30 mg Fe/mL; AMAG Pharmaceuticals, Lexington, MA, USA) were obtained from a pharmacy or directly from the manufacturer.

### 2.2 Cryo-scanning transmission electron microscopy (cryo-STEM)

The cryo-STEM studies were performed at a TFS Talos F200× (Thermo Fisher Scientific, USA) operated at 200 kV of acceleration potential. The Gatan cryo-(S)TEM holder (626 single tilt cryo-transfer holder, Gatan, Pleasanton, CA, USA) was used to ensure the nitrogen-temperature environment of the specimen during the studies. The STEM probe of about 0.25 nm in diameter (convergence angle 10.5 mrad, probe current 0.3 nA) was scanned over the region of a specimen and enabled imaging and analyses of the specimens with high resolution. The simultaneous, time- and position synchronized acquisition of different solid angle ranges of scattered electrons was enabled by using the coaxially positioned circular bright field (BF) and annular dark field detectors (low angle annular dark field (LAADF) and high angle annular dark field (HAADF)). The STEM illumination and acquisition parameters were carefully chosen to provide physically different information gain from these detectors. Namely, at the 77 mm of the camera length, the collection angle range of circular BF STEM detector was 11 mrad, for annular LAADF and HAADF detectors was 15–68 mrad and 70–200 mrad, correspondently. This insured that BF and LAADF signals were dominated by the diffraction contrast, whereas the HAADF image presented the atomic number sensitive contrast.

Our experimental set-up did not allow for low-dose conditions analysis. Therefore, to minimize the electron beam induced change in the specimen during an image acquisition, we worked in the under-sampling mode (the probe size smaller than the pixel size). Since the electron dose measurements in STEM is a highly non-trivial task for the estimation of the beam damage threshold [25], we performed the preliminary, material volume sacrificing scans of a few specimen regions while visually observing the behavior of an image feature while illuminating the region. Upon these scans, we adjusted the pixel size (magnification and frame size), the probe dwell-time (time spent by the probe on a position), probe current and probe size so not to induce visually detectable modifications of the image feature. Then, we applied the chosen parameters as valid to the data collection for a given specimen. We performed such procedure of refining the imaging parameters for each specimen prior to acquiring the data.

For the TEM specimens of all materials in study, the following parameters guaranteed no visually detected beam damage: for 1024 × 1024 K micrographs acquired at the 630 000 times (630kx) magnification (the pixel size = 0.18 nm) and with the 3 μs of the dwell time, the time for manual focusing of a specimen area was maintained under 2 s. An example of a beam damage induced effects on a material in the STEM beam illuminating the region for extended period of time is presented in Fig. S3. The image sequence shows the “on-line” evolution of the specimen volume in the beam evidencing the coalescence of the nanoparticles in a cluster and densification of the cluster upon longer illumination of the volume.

### 2.3 Cryo-transmission electron microscopy (cryo-TEM)

Cryo-TEM studies were performed on a TFS Tecnai F20 operated at 200 kV of acceleration potential and using the cryo set-up. The data were acquired with a Falcon II 4K, the direct electron detector camera. The advanced cryo-TEM work was carried out on a TFS Titan Krios (Thermo Fisher Scientific, USA) operated at 300 kV of acceleration voltage and equipped with a Gatan Quantum LS Energy Filter (GIF) and a Gatan K2 Summit direct electron detector (Ametek Pleasanton, USA). The data were acquired in an energy filtered TEM (EFTEM) operation mode using the TFS EPU software (130 000 times magnification, ∼40 e/Å^2^ total electron dose, K2 camera in a linear mode, within the 2–4 μm defocus range). The vitrified specimens were loaded into a cryo-stage of the Krios and constantly kept at 80K during the measurements.

### 2.4 Sample preparation

Specimen preparation for cryo-STEM and cryo-TEM was done in a Vitrobot Mark IV plunge freezer (Thermo Fisher Scientific, USA) at 22 °C and 100 % humidity. About 3 μL of a material was placed on Quantifoil R2/2 holey carbon TEM Cu-grids (Quantifoil, Germany), coated by a continuous 2 nm amorphous carbon layer. Prior to deposition of the material on the grids, the latter were hydrophilized by negative glow discharge (Emitech K100×, GB). Then, the grids were blotted automatically for 3 s to remove the excess of the liquid and immediately plunged into a mixture of liquid ethane/propane kept at 80 K. The TEM grids with a thin layer of the vitrified material were stored in a container placed in liquid nitrogen until they were mounted into the cryo-(S)TEM holder immediately before the TEM/STEM studies. This specimen preparation route was established and optimized over a long period of employing alternative strategies considering the material amount, type of TEM supporting grids, duration and the sign of the glow-discharge, duration of blotting, etc. as the parameters of the optimization. The optimized specimen preparation route was then used for each material in study for each TEM/STEM experiment. Particular care was taken to maintain all the specimen preparation steps and parameters unchanged, which guaranteed a uniform thinness (under 100 nm) and mechanical stability of the vitrified layer and guarantied the same study conditions for all the materials. For the automated specimen loading procedure for cryo-TEM studies, the vitrified grids were clipped into AutoGrid specimen carriers (Thermo Fisher Scientific, USA). No chemicals other than the undiluted materials from the various manufacturers and pharmacies were used. Unopened vials were accessed with a syringe to obtain the amount of the material for grid preparation.

### 2.5 Images analysis

The interactive machine learning for (bio)image analysis program Ilastik [36] was used to train cluster segmentation models. For each product, several TEM/STEM images were used to iteratively train and improve a product-specific cluster segmentation model and then applied to the whole data set for the morphological analysis. The objective during model training and tuning was to accurately capture the mass (all adherent pixels) of both clusters and individual iron cores (clusters are composed of multiple iron cores). The segmented images (5–20 per product) acquired at the same magnification were post-processed to get rid of the objects near image edges, to fill the holes in the segmented objects, and filter out segmentation noise. Properties of the final segmented clusters and individual iron cores, such as size and ellipticity, were analyzed using the scikit-image python library [37].

To verify if the STEM data are suitable for training a neural network model that can distinguish between different iron products in a self-supervised manner, we used a recently developed anomaly detection network model, PANDA [38]; trained it on FCM data and then applied the trained model to the large data sets for all materials in study. As a starting point PANDA uses a large ResNet [39] network pre-trained on the auxiliary task of ImageNet [40] dataset classification. Networks trained on auxiliary tasks (or datasets) usually require adaptation before being used for anomaly scoring on the target data. Therefore, in the PANDA model the ResNet network is fine-tuned on the training data set (here the FCM data set) with the elastic weight consolidation method [41], which maximizes the separation of standard from anomalous images. Finally, a scoring function, based on the features extracted by processing images with the fine-tuned network, is used to assign an image anomaly score. The PANDA's scoring function uses the distance from the features of the k-Nearest-Neighbour normal (FCM) images. Ideally, Cryo-STEM images of products different than FCM should get high PANDA scores.

### 2.6 Dynamic light scattering (DLS), zeta potential and pH measurements

A Malvern Zetasizer Nano ZS (ZEN3600) equipped with Zetasizer software 7.12, Helium Neon laser (633 nm, max. 4 mW) and 173° backscattering geometry was used to determine the z-average particle diameter, polydispersity index (PDI) and zeta potential. For particle size and zeta potential measurements disposable plastic cuvettes (LLG labware, LLG-Küvetten, Makro, PS, 4 ml) and a folded disposable capillary cell (DTS1070, Malvern) were used, respectively. For the accuracy testing of the instrument a 20 nm diameter NIST™ traceable size standard (3000 Series Nanosphere, Thermo Fisher Scientific, 3020A) and zeta potential transfer standard (Malvern, No. ZTS1240), were used. For particle size measurements samples were diluted in deionized water, and for the zeta potential measurements samples were diluted in deionized water and the pH value was adjusted to pH 7.4 ± 0.1 using diluted sulfuric acid or sodium hydroxide solution. A benchtop pH-meter was used to measure the pH of the samples.

## 3 Results

### 3.1 Cryo-STEM analysis of iron-carbohydrate complexes

**Fig. 1:**
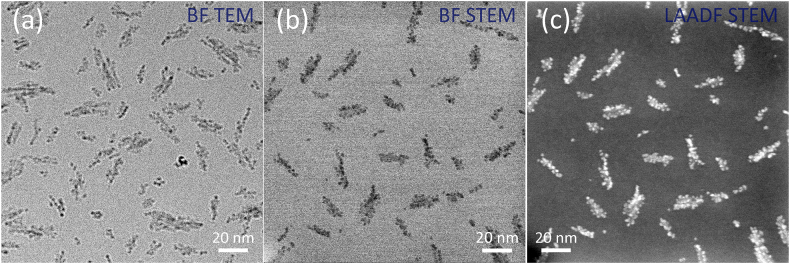
The bright-field (BF) cryo-TEM (a), bright-field (BF) cryo-STEM (b), and LAADF cryo-STEM (c) images of the ferric carboxymaltose (FCM).

**Fig. 2:**
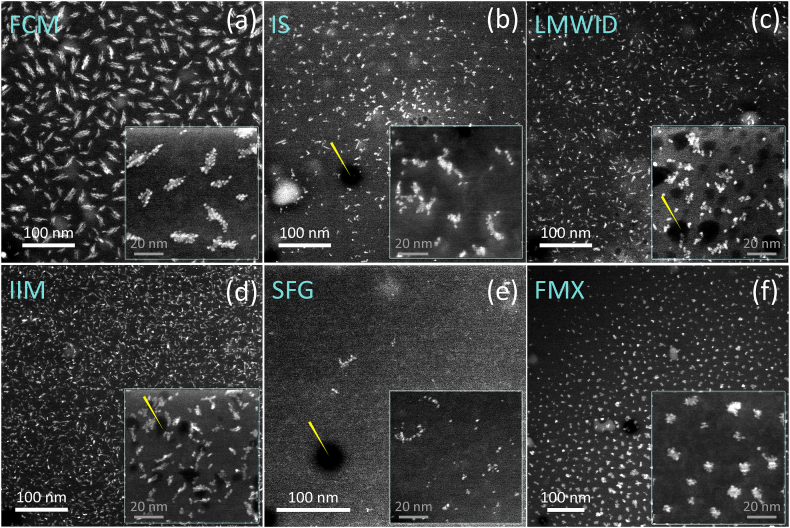
The low angle annular dark field (LAADF) cryo-STEM micrographs of (a) ferric carboxymaltose (FCM), (b) iron Sucrose (IS), (c) low molecular weight iron dextran (LMWID), (d) iron isomaltoside 1000 (IIM), (e) sodium ferric gluconate (SFG), and (f) ferumoxytol (FMX) products. The insert in the micrographs presents the data acquired at the higher magnification (630x magnification, pixel size 0.18 nm). The dark background of an about 70 nm thick amorphous layer (consisting of low-atomic number atoms and residing onto a 2 nm thick amorphous carbon substrate) provides a high contrast to the bright, crystalline clusters composed of Fe-containing nanoparticles. The background also occasionally contains foggy brighter regions (see the left bottom part of (b)) corresponding to the thickness or density fluctuations of the vitrified amorphous layer. The electron beam induced damage to the vitrified supporting layer is seen as still darker regions in the micrographs (see yellow arrows in (b, c, d, e). The micrographs are acquired in the same illumination conditions (pixel size in (a), (b), (c) and (d) is 0.49 nm, in (e) 0.7 nm and in (f) 0.35 nm) from undiluted materials.

Comparison of the cryo-TEM and cryo-STEM data acquired from the vitrified FCM product demonstrates the difference in the corresponding image contrasts. Fig. 1a and b shows the bright field TEM (BF TEM) and bright field STEM (BF STEM) micrographs, while Fig. 1c presents the data acquired using the low-angle dark field STEM (LAADF STEM) detector. The much higher contrast of the clusters in the later image is evident. Therefore, for the image analyses we used the LAADF STEM signal (Fig. 2), though for the overall morphological characterization of the materials we also used the concurrently acquired BF STEM and high-angle annular dark field (HAADF STEM) signals. For completeness, we included the BF STEM (Fig. S1) and HAADF STEM (Fig. S2) data sets from all six materials in the Supporting Information.

Fig. 2(b and c,d,e) also demonstrates that electron radiation during data acquisition could affect the structural integrity of the vitrified layer containing the clusters (see yellow arrows pointing to the decomposed volumes in the layers), though the effect of the electron beam was different for different materials: the FCM and FMX are more resilient against radiation damage.

### 3.2 Morphological and physicochemical analysis of iron-carbohydrate complexes

The manual assessment of the morphology of the iron-carbohydrate complexes (iron core sizes) was performed using 1024 × 1024 K LAADF STEM micrographs acquired at the 630kx and employing the Line Profile routine of the GMS3.21 package (Gatan Inc., USA). The analysis shows the iron core sizes of various iron-carbohydrate complexes. Furthermore, we assessed additional physicochemical attributes, such as particle diameter, zeta potential, pH, and iron concentration (see Table 1).

**Table 1:** Morphological and physicochemical characteristics of ferric carboxymaltose (FCM), iron sucrose (IS), low molecular weight iron dextran (LMWID), Iron isomaltoside (IIM), Sodium ferric gluconate (SFG), and Ferumoxytol (FMX).

Material	Cryo STEM^a^: area of the objects, (nm^2^^b^)	Cryo-STEM^b^: Fe-core size <d>, (nm)	DLS Z-Average Diameter (nm)	DLS Polydispersity index (PDl)	Zeta Potential at pH 7.4, (mV)	pH^c^	Elemental Fe Concentration, (mg/mL)^d^
FCM	122 ± 96	1.8 ± 0.18	24.5	0.07	−10	5.8	50
IS	24 ± 30	1.9 ± 0.18	11.4	0.13	−34	10.9	20
LMWID	21 ± 21	2.0 ± 0.18	16.1	0.08	−22	6.1	50
IIM	19 ± 19	1.6 ± 0.18	14.3	0.14	−30	5.9	100
SFG	16 ± 26	1.4 ± 0.18	14.2	0.17	−34	8.4	12.5
FMX	39 ± 29	1.8 ± 0.18	34.3	0.18	−49	7.4	30

a Ilastik software analysis.

b Manual morphological assessment using LAADF STEM data. The assessments are performed using a limited number of clusters in a few images (per product). The one-pixels width, 0.18 nm, is taken as a standard deviation.

c pH of the dispersion.

d Per each products approved label.

### 3.3 Self-supervised machine learning for image analysis of iron-carbohydrate complexes

**Fig. 3:**
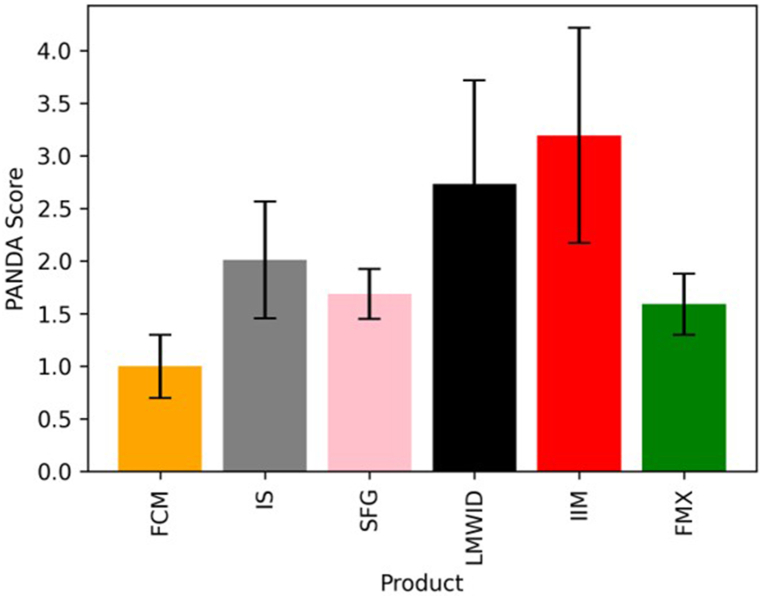
Analysis of STEM micrographs with the PANDA model trained on the ferric carboxymaltose (FCM) micrographs. The mean normalized PANDA scores and standard deviations are shown for each product. Statistics are calculated over the product micrographs. Classifier scores are normalized to the mean of the FCM test set score.

We used a self-supervised machine learning method for image analysis of the different iron-carbohydrate complexes. The trained model was run on the data sets of all six products to deliver the characteristic (or “clear-cut”) classifier value for each STEM micrograph of all the materials. The number of micrographs used for the analysis was: 19 for FCM (images not selected for the training were used for the analysis), 29 for IS, 4 for IIM, 12 for FMX, 10 for LMWID, and 8 for SFG, which is all the images collected for the products at the 630kx magnification. Fig. 3 presents the normalized PANDA classifier scores for the analyzed products.

## 4 Discussion

Intravenous iron-carbohydrate complexes are widely used therapeutic agents for the treatment of iron deficiency and iron deficiency anemia in a diverse range of patient populations. However, a full understanding of their physical structure and its criticality for their behavior especially at the nano-bio interface is still lacking, highlighting the need for additional data and advanced orthogonal characterization techniques.

This study utilized cryo-STEM to investigate the morphology of iron carbohydrate nanoparticles, aiming to bridge the knowledge gap in this field. This technique builds upon the work conducted internally at FDA [32], where they employed cryo-TEM to provide direct visualization of the iron cores in these complexes. By expanding on these methods and results, we advanced the understanding of the physical structure of the iron-carbohydrate nanoparticles *in situ* by examining the presence of cluster-like structures across various iron-carbohydrate products.

The application of cryo-STEM in this study proved to be a more robust and sophisticated approach for characterizing the cluster-like structures of iron carbohydrate nanoparticles within the finished product in its pristine state. These typical cluster structures of the distinct iron-carbohydrate products may explain the previously observed size differences between cryo-TEM and DLS data [13,32]. A recent study using Small-angle X-ray scattering (SAXS) and Small Angle Neutron scattering (SANS) further confirmed our findings [34]. The cryo-STEM technique offers high contrast darkfield imaging capabilities, enabling precise visualization of the nanoparticles, and the ability to analyze samples at cryogenic temperatures, importantly preserving their structural integrity. This combination of imaging and analytical capabilities makes cryo-STEM an additional valuable tool for more comprehensive characterization of these nanoparticles.

### 4.1 Cryo-TEM versus cryo-STEM

With the purpose of establishing the most accurate and reliable TEM characterization technique for the comparative studies of different iron-carbohydrate complexes, we performed TEM analyses of the same material by two different operation modes: cryo-TEM and cryo-STEM. The first employs a parallel electron beam illuminating a region of the vitrified material and provides the BF TEM data relying on the diffraction contrast of the crystalline component of the complexes and on the mass-thickness contrast from the specimen regions composed from different atomic species. This broadly used method for TEM analyses gives the dark grey against the bright grey contrast in the images. The second, STEM operation mode uses a sharp converged probe scanning across a region of the vitrified specimen and yields BF STEM data. In addition, STEM also provides the complementary, high contrast signals from coaxial detectors: the BF STEM and LAADF STEM data provide mainly the diffraction contrast signals from crystalline component of a cluster, whereas the HAADF STEM micrographs show the strong atomic number contrast of the Fe-containing cores against the low atomic number of the vitrified layer material and the carbohydrate component of the system.

The scattering angle ranges of the incident electrons carry the information about different mechanisms of the electron - matter interaction. Low-angle electron scattering is dominated by diffraction effects of the electrons on specimen atoms due to Bragg scattering. Whereas high-angle scattering carries the information about the atomic number of atoms; the greater the atomic mass, the more pronounced the proximity of the incident electron's trajectory to the atomic nucleus, thereby increasing the scattering angle due to the enhanced Coulombic attraction to the nucleus [35,42].

When a fine electron probe of STEM illuminating a very small volume of the specimen at a given position moves pixel by pixel and line by line over the specimen region, the annular STEM detectors generate “brightness maps” of the region, where the high angle annular dark field (HAADF) image shows the distribution of different atomic species in the scanned region (i.e. presenting the so-called “Z-contrast”) and the low angle annular dark field (LAADF) detector presents the signal combining both, weak atomic number contrast added to the very prominent diffraction contrast from crystalline volumes in the region. For completeness, the BF STEM detector, a circular detector positioned co-axially on the optical axis of an instrument and registering the lowest angle range of scattered electrons, presents the “brightness map” of the region dominated by the diffraction contrast added to the weak signal of the mass-thickness contrast in the specimen volume [35]. Incidentally, the latter, the mass-thickness signal of the BF STEM detector is basically the same as obtained in the BF TEM image signal of the cryo-TEM setup. The complementary information of the LAADF signal (“diffraction contrast” plus “atomic number contrast”) turned out to be very beneficial for imaging of very small, Fe-containing crystalline volumes embedded in the vitrified support layer of the specimen.

Since the physical origins of the BF TEM and BF STEM signals are the same, the mass-thickness contrast and the diffraction contrast, the BF TEM (Fig. 1a) and BF STEM (Fig. 1b) data present very similar images of the clusters embedded in the vitrified amorphous layer. The difference between the BF TEM and BF STEM is due to the different focus of the features. To enhance the weak contrast from small crystallites, the cryo-TEM micrographs are normally acquired at a certain defocus (see Fig. 1a), whereas the cryo-STEM data are accurately focused by setting a very sharp converged probe to a particular plane within the specimen thickness. Accurate focusing gives an immediate advantage to the BF STEM data providing reliable morphological information of the clusters. Next, the dark field STEM imaging provides yet stronger contrast (compare the BF STEM (Fig. 1b) to the LAADF STEM (Fig. 1c) data): the bright signal of the crystalline, iron-containing clusters against the dark background of the amorphous support layer (Fig. 1c) is obviously stronger than the dark grey against bright grey signal of the BF STEM data (Fig. 1b). Therefore, the LAADF STEM data are optimally suited for the automation of the image evaluation procedures.

### 4.2 Cryo-STEM analysis of iron-carbohydrate complexes

Considering both, accurate focusing and the high contrast of STEM data, we predominantly used the cryo-STEM operation mode for the manual and automated image evaluations routines during the comparative analyses of different iron-carbohydrate complexes. Fig. 2 presents the LAADF STEM data on the vitrified undiluted specimens of all six products analyzed at the same illumination and acquisition parameters. Simple visual inspection of the micrographs from different materials showed that the clusters of different products have differing morphologies. The FCM (Fig. 2a) and FMX (Fig. 2f) materials show the most clearly defined cluster conformations of all six products. The elongated clusters of FCM are composed of strands of easily discernible crystalline iron cores, while the clusters of FMX are very dense and nearly equidimensional. Very small volume fractions of single NPs (composed of only one iron core) are present in both products. During the STEM measurements, both materials always showed a very homogeneous cluster distribution in the vitrified layer. Incidentally, we found that these layers were the most stable among all six products against electron radiation: at the same illumination and acquisition conditions, these materials sustained beam exposure without any detectable structural damage longer than other materials. However, we cannot provide any quantified information to substantiate the observations at this stage of the studies. This made the analyses of FCM and FMX materials the easiest for manual assessment and the most reliable and accurate for the automated image evaluation workflow.

The LMWID (Fig. 2c) and IIM (Fig. 2d) possess a broader range of the cluster shapes. They also show the presence of single NPs, though still with a clear predominance of the volume fraction of the clusters. Between the two products, the LMWID clusters are denser, and their shapes are better established though more diverse. The IIM clusters are generally more elongated and less compact.

The IS (Fig. 2b) and SFG (Fig. 2e) materials have the broadest range of the cluster morphologies and possess the largest volume fractions of single NPs. Generally, SFG exhibits the least compact morphology of the clusters and the largest number of single NPs of all the products examined.

### 4.3 Morphological and physicochemical analysis of iron-carbohydrate complexes

The manual assessment of the morphology shows that FCM and FMX contain iron core sizes of approximately 1.8 nm, while LMWID, IIM, IS, and SFG exhibit iron core sizes of about 2.0 nm, 1.6 nm, 1.9 nm, and 1.4 nm, respectively (see Table 1). It is important to mention that the manual morphological assessment using LAADF STEM data (see Fig. S4) is performed using a limited number of clusters in a few images per product and cannot be considered statistically representative. However, the results are consistent with previous observations by Y. Wu et al. [32], where the same materials, prepared by preserving the iron-carbohydrate complexes in their native, frozen-hydrated, and undiluted state, revealed iron cores of about 2 nm in size.

The high contrast and resolution of the LAADF signal of cryo-STEM provided a high-quality data set such that tedious manual assessment of the cluster morphologies of the products could be easily assisted and even substituted by automated image analysis. The latter was conducted utilizing the Ilastik software and provided most reliable statistical analysis and high-resolution information on the cluster morphologies. To avoid the ambiguity in evaluation due to the anisotropic shape of clusters in some products and, consequently the problems with the comparative characterization of the products, the “area” of the objects was chosen as a characteristic evaluation parameter (see Table 1) rather than the diameter or length. These values deliver the smaller cluster dimensions (even assuming the isotropic cluster morphology) when compared to the hydrodynamic diameter of the clusters provided by DLS measurements because the latter relate to the size of the clusters including the carbohydrate ligands.

The DLS z-average diameters ranged from 11.4 to 34.3 nm, with polydispersity indexes (PDI) ranging from 0.07 to 0.18 are in line with the observations reported by Jahn et al. [13]. As expected, the hydrodynamic sizes of the iron-carbohydrate nanoparticles exceed the iron core sizes determined via cryo-STEM, consistent with the cluster-like morphology observed in all examined products. All six iron-carbohydrate complexes investigated exhibit a negative charge, spanning from −10 mV to −49 mV, with pH values ranging between 5.8 and 10.9. The iron concentration was not directly measured but obtained from the product labels.

### 4.4 Self-supervised machine learning for image analysis of iron-carbohydrate complexes

Relying on the convincing evidence of the morphological distinction of the iron-carbohydrate complexes of different products studied in our work, we attempted to verify if the cryo-STEM data can be used for training a neural network model that can discern the iron-carbohydrate complex materials in a self-supervised manner. We speculate that a trained model should be able to recognize and identify the cluster characteristics directly from the cryo-STEM data.

For this purpose, we employed a recently developed pre-trained anomaly detection network model, PANDA [38] that uses features of a given dataset to train image classifier and then utilizes this pre-trained solution to the analyses of further data sets. Thus, in our work we exposed the neural network model to a fraction of the data on the FCM (we used 48 LAADF cryo-STEM micrographs in the training set). No data on other products were used during the classifier training. The classifier training process converged after 20 epochs.

The AUC-ROC (Area Under the Curve of the Receiver Operating Characteristic) value for FCM micrographs versus that for the data of the rest of the products is equal to 0.98, which is close to a perfect separation since the distribution of PANDA scores (FCM vs other products) is practically non-overlapping (see Fig. 3). The seemingly low number of the micrographs that was used to train the accurate classifier can be attributed to the clear morphological differences between the analyzed products. The classification model could be further developed into a useful characterization tool for comparability studies trigged by manufacturing changes.

## 5 Conclusions

Consistent with previous observations by Y. Wu et al. [32], our findings confirm that the average size of the iron cores in these nanoparticles is approximately 2 nm. This size range was consistent across all investigated iron-carbohydrate products. Moreover, our study revealed the presence of distinct cluster-like morphologies not only associated with FMX, as previously reported, but among all iron-carbohydrate products investigated. Furthermore, we utilized a self-supervised machine learning method for image analysis, which has the potential to be further developed into a valuable characterization tool for comparability studies triggered by manufacturing changes, for example.

The data obtained from this study advances the understanding of the physical structure of the iron-carbohydrate nanoparticle preparations in their pristine state. Specifically, by analyzing samples at cryogenic temperatures the structural integrity of the iron-carbohydrate nanoparticles can be better preserved and a more accurate understanding of their 3-dimensional structure can be achieved. This finding can accelerate future studies to further elucidate the complex structure-function relationship of this widely used drug class. Adding this sophisticated cryo-STEM technique to the battery of emerging, and established techniques for physicochemical characterization of iron-carbohydrate nanoparticles will improve understanding of their 3-dmensional physical structure. Ultimately, these techniques will facilitate a more accurate and comprehensive understanding of the link between manufacturing, product physicochemical characteristics, and their *in vivo* behavior at the nano-bio interface.

## Funding

The work was funded by CSL Vifor.

## Data availability statement

Data was included in the article, support material and referenced in article.

## CRediT authorship contribution statement

**Reinaldo Digigow:** Writing – original draft, Conceptualization. **Michael Burgert:** Conceptualization. **Marco Luechinger:** Conceptualization. **Alla Sologubenko:** Writing – original draft, Methodology, Formal analysis, Data curation. **Andrzej J. Rzepiela:** Writing – original draft, Methodology, Formal analysis, Data curation. **Stephan Handschin:** Writing – original draft, Methodology, Formal analysis, Data curation. **Amy E. Barton Alston:** Writing – review & editing. **Beat Flühmann:** Writing – review & editing. **Erik Philipp:** Writing – review & editing, Conceptualization.

## Declaration of competing interest

The authors declare the following financial interests/personal relationships which may be considered as potential competing interests:Alla Sologubenko, Andrzej J. Rzepiela, Stephan Handschin reports financial support was provided by Vifor (International) AG. Reinaldo Digigow reports a relationship with Vifor (International) AG that includes: employment. Michael Burgert reports a relationship with Vifor (International) AG that includes: employment. Marco Luechinger reports a relationship with Vifor (International) AG that includes: employment. Amy E. Barton Alston1 reports a relationship with Vifor (International) AG that includes: employment. Beat Fluhmann reports a relationship with Vifor (International) AG that includes: employment. Erik Philipp reports a relationship with Vifor (International) AG that includes: employment. If there are other authors, they declare that they have no known competing financial interests or personal relationships that could have appeared to influence the work reported in this paper.
