# Supplementary material for: Nano-scale characterization of iron-carbohydrate complexes by cryogenic scanning transmission electron microscopy: Building the bridge to biorelevant characterization
Source: Heliyon. 2024 Aug 24;10(17):e36749. doi: 10.1016/j.heliyon.2024.e36749 (PMC11401109; doi:10.1016/j.heliyon.2024.e36749)
Supplement: Multimedia component 1 [file mmc1.docx]

**Supporting Information**

**Nano-scale characterization of iron-carbohydrate complexes by cryogenic Scanning Transmission Electron Microscopy: building the bridge to biorelevant characterization**

*Reinaldo Digigow^1^, Michael Burgert^1^, Marco Luechinger^1^, Alla Sologubenko^2^, Andrzej J. Rzepiela^2^, Stephan Handschin^2^, Amy E. Barton Alston^1^, Beat Flühmann^1*^, Erik Philipp^1^*

1. CSL Vifor, Flughofstrasse 61, CH-8152 Glattbrugg, Switzerland
2. Scientific Center for Optical and Electron Microscopy, ScopeM, ETH Zürich, 8093 Zürich, Switzerland

*Corresponding author:

E-mail address:

[Beat.fluehmann@viforpharma.com](mailto:Beat.fluehmann@viforpharma.com)


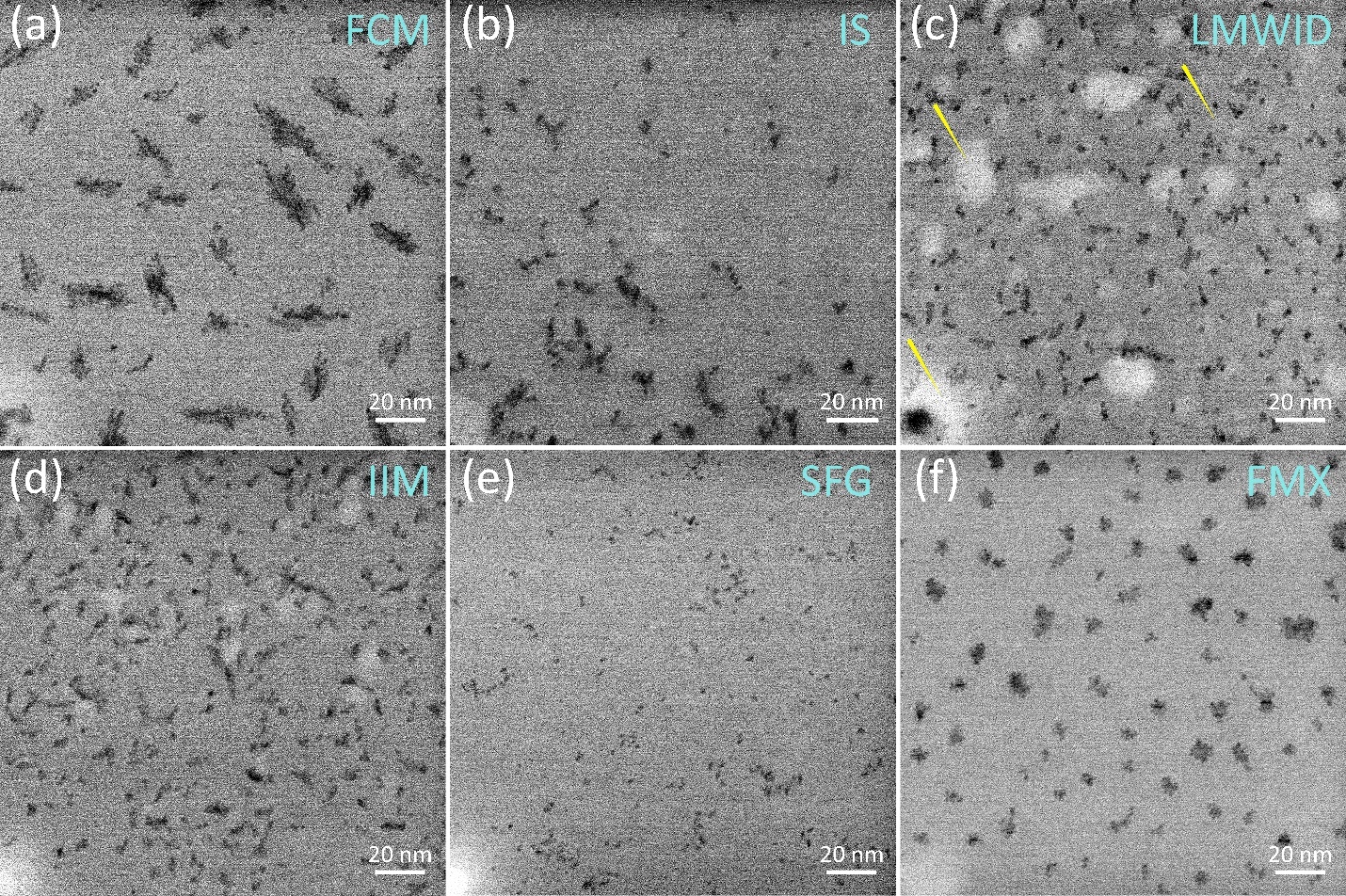


**Fig. S1.** Cryo BF STEM data of (a) ferric carboxymaltose (FCM), (b) iron Sucrose (IS), (c) low molecular weight iron dextran (LMWID), (d) iron isomaltoside 1000 (IIM), (e) sodium ferric gluconate (SFG), and (f) ferumoxytol (FMX) materials. The micrographs are acquired concurrently with the data presented in Fig.2 and show the same specimen regions. The small dark nanoparticles within the clusters can be clearly resolved in each image, though the contrast in the micrographs is weaker than that in LAADF STEM data (Fig.2). All micrographs are acquired in the same illumination conditions and at the same magnification (pixel size is 0.18 nm) – undiluted samples.


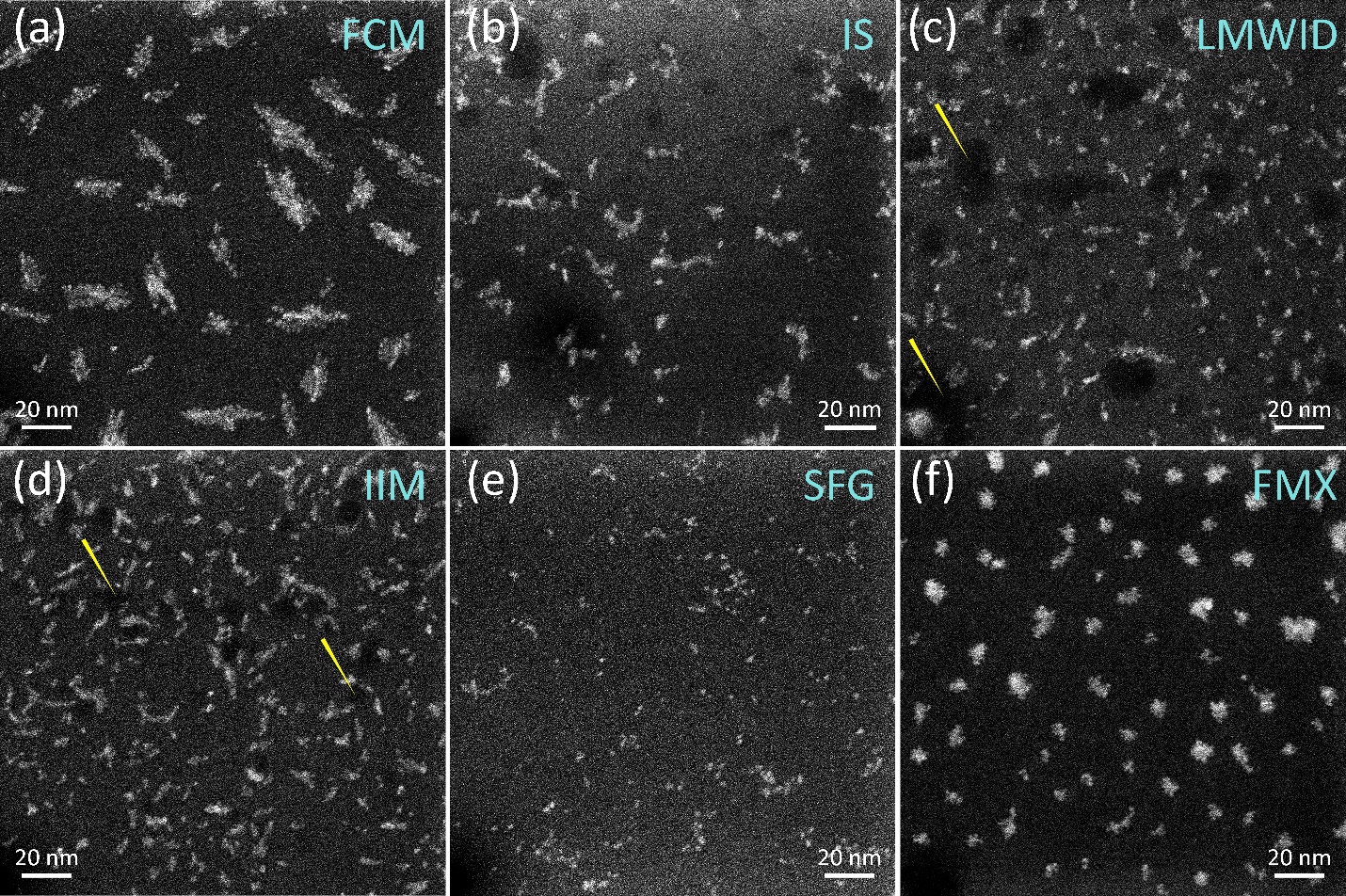


**Fig. S2.** Morphological cryo-STEM analyses of (a) ferric carboxymaltose (FCM), (b) iron Sucrose (IS), (c) low molecular weight iron dextran (LMWID), (d) iron isomaltoside 1000 (IIM), (e) sodium ferric gluconate (SFG), and (f) ferumoxytol (FMX) materials, acquired using the diffraction contrast sensitive HAADF STEM detector. The micrographs are acquired concurrently with the data presented in Fig.2 and show the same specimen regions.


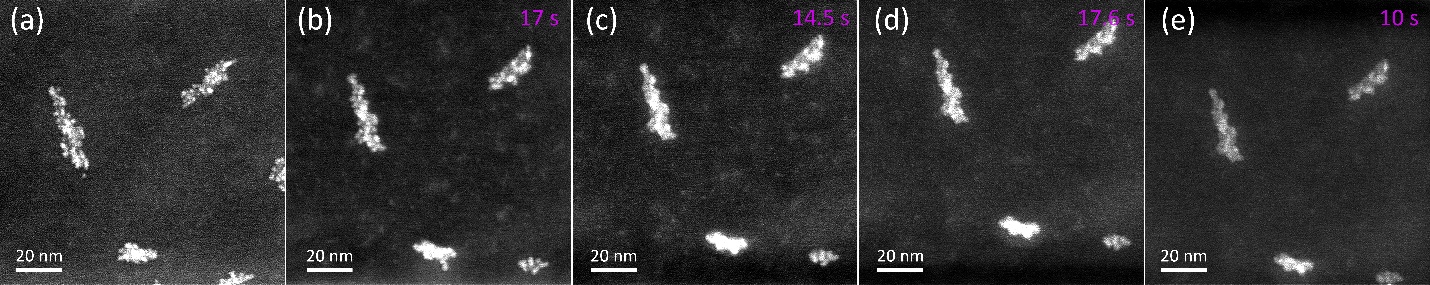


**Fig.S3.** Electron beam induced damage of a ferric carboxymaltose (FCM) material. The LAADF STEM image sequence is acquired in a movie-like fashion and demonstrates the material alteration and degradation due to an electron beam exposure. The numbers in (b)-(e) present the time interval (in seconds) between the acquisitions of two consecutive images. The pixel size = 0.25 nm, frame size = 512 x 512, frame time = 630 msec.


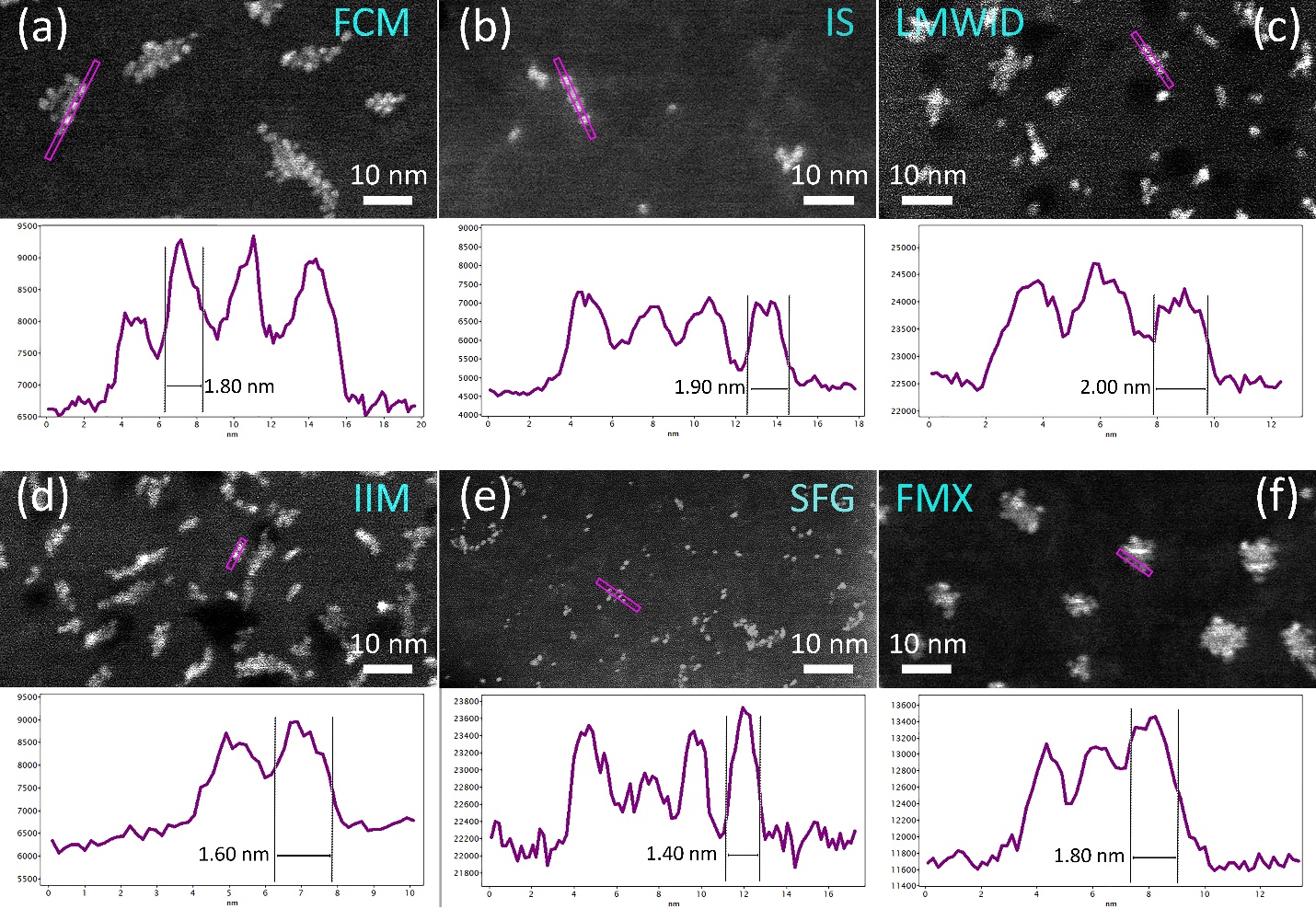


**Fig.S4.** Cropped-out frames of the micrographs in Fig.2 (cryo LAADF STEM) show the complex internal structure of the clusters and the corresponding intensity profiles along the chains of the iron core nanoparticles within a cluster. The profiles present the results of a manual assessment of the nanoparticle size and demonstrate the difference between the core sizes of different materials. The ultimate accuracy of the manual estimation of the nanoparticle size is limited by the image pixel size, which is 0.18 nm for all micrographs.

**Table S1*:** Iron-carbohydrate complexes.

| **Generic name** | **Ligand(s)** | **Ligand Description** |
| --- | --- | --- |
| Iron Sucrose (IS) | Sucrose | Disaccharide |
| Sodium Ferric Gluconate (SFG) | Gluconate and sucrose | Carboxylic acid and disaccharide |
| Low Molecular Weight Iron Dextran (LMWID) | Dextran | Polysaccharide, maltose units 1→6-linked |
| Iron Isomaltoside 1000 (IIM) | Isomaltoside 1000/derisomaltose, and citrate | Oligosaccharide, maltose units 1→6-linked, hydrogenated, and carboxylic acid |
| Ferric Carboxymaltose (FCM) | Carboxymaltose | Polysaccharide, maltose units 1→4-linked, oxidized |
| Ferumoxytol (FMX) | Polyglucose sorbitol carboxymethyl ether and mannitol (excipient) | Polysaccharide, maltose units 1→6-linked, hydrogenated and carboxymethylated, and monosaccharide |

^*^ Adapted from Funk F, Fluhmann B, Barton AE. Criticality of Surface Characteristics of Intravenous Iron-Carbohydrate Nanoparticle Complexes: Implications for Pharmacokinetics and Pharmacodynamics. Int J Mol Sci. 2022;23(4)

**Table S2:** Number of objects on the analyzed images**.**

| **Product** | **Nr of images** | **Total Nr of objects** | **Mean nr of objects per image** | **Std of objects per image** |
| --- | --- | --- | --- | --- |
| FCM | 48+19 | 2659 | 39.7 | 8.1 |
| IS | 29 | 1074 | 37.0 | 23.4 |
| SFG | 8 | 465 | 58.1 | 59.0 |
| LMWID | 10 | 1815 | 181.5 | 44.3 |
| IIM | 4 | 989 | 247.2 | 18.2 |
| FMX | 12 | 476 | 39.7 | 12.2 |
